# Oxygen extraction fraction is differentially associated with pathological biomarkers in Alzheimer’s disease and non-Alzheimer’s dementias

**DOI:** 10.3389/fnins.2026.1754415

**Published:** 2026-04-28

**Authors:** Arpita Misra, Yi Wang, M. Reza Taheri, Gloria C. Chiang, Junghun Cho

**Affiliations:** 1 Department of Biomedical Engineering, George Washington University, Washington, DC, United States; 2 Department of Radiology, Weill Cornell Medicine, New York, NY, United States; 3 Department of Biomedical Engineering, Cornell University, Ithaca, NY, United States; 4 Department of Radiology, George Washington University, Washington, DC, United States

**Keywords:** Alzheimer’s disease, dementia, oxygen extraction fraction, QSM+qBOLD, quantitative blood oxygenation level-dependent imaging, quantitative susceptibility mapping, white matter hyperintensity

## Abstract

**Introduction:**

We aimed to understand the pathophysiological differences between 16 Alzheimer’s disease (AD) and 15 non-AD dementia patients by quantifying oxygen extraction fraction (OEF) in cortical (CGM) and deep gray matter (DGM) regions.

**Methods:**

To achieve this, we used a novel MRI-based OEF mapping technique, QQ, which estimates OEF from routine multi-echo gradient echo data. Multiple linear regression analyses were performed to compare the associations between OEF and white matter hyperintensities (WMH) or cognitive impairment (measured by Montreal Cognitive Assessment (MoCA) between the two groups.

**Results:**

In the AD and non-AD group, OEF showed negative associations with WMH in DGM and positive associations with MoCA in DGM and CGM.

**Discussion:**

Our study suggests that QQ is a promising tool for differentiating between AD and non-AD dementias, by revealing abnormalities in tissue oxygen usage and their relationships to microvascular changes and cognitive impairment.

## Introduction

Dementia is a persistent cognitive impairment that predominantly affects older individuals (Arvanitakis et al., 2019). Alzheimer’s disease (AD) is the most common form of dementia, characterized by the accumulation of beta-amyloid peptides in extracellular plaques and hyperphosphorylated tau protein within intracellular neurofibrillary tangles, leading to synaptic loss and neuronal dysfunction (Ballard et al., 2011; Brion, 1998; Goedert et al., 1991; Rogan and Lippa, 2002; Shoghi-Jadid et al., 2002). On the other hand, almost 20–40% of dementia cases are non-AD (Alzheimer’s and Dementia, 2023), with pathology and progression distinct from AD (Lovestone, 2009; Rogan and Lippa, 2002).

Unfortunately, most MR biomarkers for dementia have shown similar changes in both AD and non-AD cases (Rosenberg et al., 2016). Differentiating between AD and non-AD dementias is particularly challenging due to significant overlap in clinical symptoms, such as initial memory impairment and cognitive decline (DeTure and Dickson, 2019). Moreover, biomarkers such as tau tangles, while hallmarks of AD, can also appear in other forms of dementia (Iseki et al., 2006), limiting their specificity. This ambiguity underscores the need for advanced techniques capable of distinguishing between AD and non-AD dementias, thereby enabling tailored therapeutic interventions.

Oxygen extraction fraction (OEF)—the percentage of oxygen that tissue extracts from blood and serves as a direct biomarker for tissue viability and functionality (Biondetti et al., 2023; Jiang and Lu, 2022; Kudo et al., 2015)—may provide new insights into the causes and progression of different types of dementia. For example, a longitudinal study found different associations between AD biomarkers (e.g., amyloid burden) and OEF among dementia patients with varying levels of vascular risk (Jiang et al., 2020).

Recently, a promising MRI-based OEF model, termed QQ, has been developed by integrating quantitative susceptibility mapping (QSM) (Ma et al., 2020a; Ma et al., 2020b) and quantitative blood oxygen level-dependent magnitude (qBOLD) (Cho et al., 2018, 2020b,2021a,2021d; Kudo et al., 2016) (QSM+qBOLD = QQ) (Cho et al., 2018). QQ has significant clinical potential for mapping OEF, as it utilizes a single routine sequence available on every MRI scanner, multi-echo gradient echo (mGRE) (Hubertus et al., 2019b), and does not require impractical data acquisition schemes, multiple gas inhalations or vascular challenges, unlike other OEF mapping techniques (Biondetti et al., 2023; Mintun et al., 1984; Smeraldo et al., 2022). The robustness of QQ has been further improved (Misra and Cho, 2025) by machine learning and deep learning algorithms (Cho et al., 2020b,2021d,^2022^, ^2024^; Hubertus et al., 2019c).

The QQ model has been validated against ^15^O-PET^21^ and has demonstrated sensitivity to physiologically induced increases (via hyperventilation) and decreases (via hypercapnia) in OEF levels in healthy subjects (Cho et al., 2021b; Elanghovan et al., 2024). It has also been clinically applied to various neurologic disorders, including ischemic stroke (Cho et al., 2019; Wu et al., 2021; Zhang S. et al., 2018; Zhang et al., 2020), pre-eclampsia (Yang et al., 2022), (Zhang et al., 2023), hydrocephalus (Zhuang et al., 2023), multiple sclerosis (Cho et al., 2020a,2021c; Vallejos et al., 2024; Xie et al., 2024), Parkinson’s disease (PD) (Misra et al., 2025a; Yan et al., 2023; Yan et al., 2025), glioma (Hubertus et al., 2019a; Shen et al., 2021; van Grinsven et al., 2019), age-related changes in normal adults (Wang et al., 2022; Wu et al., 2024) and other neurologic disorders (Feng et al., 2023; Zhang R. et al., 2025; Zhang S. et al., 2025). Although QQ has been previously used in studies with cognitively impaired patients and AD (Chiang et al., 2022; Hosseini et al., 2023; Misra et al., 2025b), differences in OEF between AD and non-AD dementias have not yet been investigated.

Building on our previous work showing OEF differences between healthy controls and AD patients, the present study investigates alterations in oxygen metabolism among cognitively impaired individuals with AD and non-AD dementia. Specifically, we compared the relationships of QQ-based OEF with dementia-related biomarkers between AD and non-AD dementia, including: (1) white matter hyperintensities (WMH; a measure of microvascular change and strong predictor of dementia (Prins and Scheltens, 2015)) and (2) cognitive performance, assessed by the Montreal Cognitive Assessment (MoCA) score.

## Materials and methods

### Study cohort

The study protocol was approved by Weill Cornell Medicine IRB review board. Written consent was provided by all the participants and/or their legal representatives. Thirty-one cognitively impaired patients who were referred for clinical care between 2017 and 2021 and had cerebrospinal fluid (CSF) biomarkers for dementia characterization were included in this analysis. CSF was drawn from patients by lumbar puncture, collected in sterile polypropylene collection tubes, and sent to Athena Diagnostics, which detects CSF proteins using enzyme linked immunosorbent assay methodology (Engelborghs et al., 2017).

All participants also underwent MRI and clinical, neurological, and neuropsychological assessments for characterization as AD versus non-AD. To confirm probable AD, we used the following cutoffs: ptau > 68 pg/ml and beta-amyloid (Abeta) 42 to total tau index < 0.8, which have been shown to have a sensitivity of 85% and specificity of 90% (Blennow et al., 2015). After detailed assessments and characterization by these CSF biomarkers, the cohort was classified into 16 individuals diagnosed with AD and 15 individuals with non-AD dementia. Microhemorrhages (MCH) (Rauchmann et al., 2020) were assessed in all patients to evaluate the presence and extent of small brain hemorrhages, which can indicate vascular damage and contribute to cognitive decline. White matter hyperintensities were visually assessed and scored using the Fazekas scale (Fazekas et al., 1987): mild (scored 1), moderate (scored 2), severe (scored 3).

Montreal Cognitive Assessment (MoCA) (Nasreddine et al., 2005) was performed for all patients to assess the disease severity. Classification into AD and non-AD was based on cerebrospinal fluid (CSF) biomarker profiles. Therefore, all individuals were classified as cognitively impaired, with diagnoses confirmed through biological evidence rather than cognitive scores alone.

### MRI protocol

All patients underwent MR imaging on a 3 Tesla scanner (Biograph mMR, Siemens Healthcare), which included a 3D T1-weighted MPRAGE sequence reconstructed at 1 mm slice thickness (TR/TE 2,300/2.27 ms with a flip angle of 8 degree), a 3D T2 FLAIR (Fluid-Attenuated Inversion Recovery) sequence reconstructed at 1 mm slice thickness (TR/TE/TI 7,600/386/2,400 ms with a flip angle of 120 degree) and a 3D mGRE sequence reconstructed at 3 mm slice thickness, TR/TE_1_ 49/6.68 ms, echo spacing 4.06 ms, number of echoes 10, in plane resolution 0.7188 mm × 0.7188 mm, pixel bandwidth 260 Hz/pixel and flip angle 15 degrees with flow compensation disabled, and parallel imaging (GRAPPA) enabled with an acceleration factor 2.

### Image processing

For QSM reconstruction, the total field was estimated from the mGRE phase data using linear fitting (Liu et al., 2013). A whole brain mask was generated using Brain Extraction Tool (BET) (Smith, 2002) and further eroded by a radius of 3 mm to mitigate susceptibility-related artifacts at the brain air interface. Phase unwrapping was performed using a graph-cut algorithm (Bioucas-Dias and Valadão, 2007) prior to background field removal. The background field was removed using the projection onto dipole fields (PDF) algorithm (Liu et al., 2011) with noise variance weighting and conjugate gradient optimization (maximum 30 iterations, tolerance = 0.1). Zero-padding of 40 voxels was applied to reduce boundary artifacts.

A spherical mean value (SMV) filter with a 5 mm radius was subsequently applied for any residual background field removal prior to dipole inversion. Local field inversion was then performed using morphology-enabled dipole inversion with automatic cerebrospinal fluid zero referencing (MEDI+0) algorithm (Liu et al., 2011; Liu et al., 2012; Liu et al., 2017; Liu et al., 2018; Wang and Liu, 2015). The optimization was solved using Gauss–Newton iterations with conjugate-gradient updates, employing L1-norm regularization (regularization factor λ = 1,000) together with an additional CSF regularization factor (λ = 100), yielding the final susceptibility map—QSM (de Rochefort et al., 2010; Liu et al., 2013)

OEF maps were computed using the QQ algorithm (Cho et al., 2018). QQ unifies two biophysics models: (1) QSM (Cho et al., 2018; Ma et al., 2020a; Ma et al., 2020b; Wang and Liu, 2015; Zhang et al., 2015; Zhang J. et al., 2018) and (2) qBOLD. Each mGRE signal has two parts, phase and magnitude. QSM uses the phase and qBOLD uses the magnitude part. QSM-based model distinguishes the susceptibility contributions of blood (based on its oxygenation level) and neural tissue on a per-voxel basis. qBOLD models the mGRE magnitude signal decay due to field variations within a voxel caused by the susceptibility difference between the blood and surrounding tissue (He and Yablonskiy, 2007; He et al., 2008; Ulrich and Yablonskiy, 2016; Yablonskiy and Haacke, 1994; Yablonskiy and Sukstanskii, 2017; Yablonskiy et al., 2013a; Yablonskiy, 1998). The balance between QSM and qBOLD in the joint QQ model was achieved using a weighting factor (*w*), which was determined by L-curve analysis (Cho et al., 2018). For the magnitude signals, macroscopic magnetic field inhomogeneities were compensated using the voxel spread function (VSF) method (Yablonskiy et al., 2013b), which treats macroscopic field variations as linearly varying within each voxel. Since the inversion of QQ involves nonconvex optimization, it is sensitive to the measurement noise. To minimize this noise sensitivity, the temporal clustering, tissue composition, and total variation (CCTV) algorithm was used in this study (Zhang et al., 2020). This CCTV effectively suppresses measurement noise propagation into OEF maps by applying temporal and tissue-type clustering and total variation regularization. By utilizing cluster-wise rather than voxel-wise optimization, the CCTV algorithm mitigates the impact of spatial resolution discrepancies between the QSM maps (which may be smoothed by spatial regularization used during dipole inversion) and the mGRE magnitude images. CSF voxels were excluded from QQ-CCTV processing (identified using R2* thresholding, R2* < 2.5 Hz) as OEF is physiologically meaningful only in vascularized tissue. OEF was shown as a percentage from 0 to 100 (unitless).

For region of interest (ROI) analysis, Freesurfer (Fischl et al., 2002) version 7.4.1 was used for ROI segmentation on the T1 weighted images. Segmentation outputs were visually inspected for anatomical accuracy and no major errors were observed. To mitigate potential partial volume effects at the boundaries of the DGM, a 1-voxel erosion was applied to suppress signal contamination from adjacent CSF or WM, ensuring that the OEF measurement predominantly reflects the target tissue. The T1w images were registered to OEF maps using the FSL FLIRT algorithm (Jenkinson and Smith, 2001; Jenkinson et al., 2002). Specifically, the registration was performed from T1 space to native OEF space (T1w→OEF). First, the T1w image was registered to the mGRE echo-combined T2*w image, which has the identical space as the OEF map. Using the resulting registration matrix, the anatomical segmentation maps (in the T1w image space) was then registered into OEF space using the nearest-neighbor interpolation option. Therefore, this registration did not alter the OEF voxel intensities used for ROI statistics. WMH masks were edited manually using itk-snap (Yushkevich et al., 2016) from the T_2_ FLAIR images by an experienced neuroradiologist (Dr. Chiang). The FLIRT registration was performed using 6 degrees of freedom and the Correlation ratio cost function. Following registration, the results for each subject were visually inspected slice by slice to ensure accurate anatomical alignment between the T1-weighted and mGRE spaces. The visual QC pass rate was 100%, with all subjects demonstrating high-quality alignment.

We used the volumetric fraction of WMH relative to the whole brain as the measure of WMH percentage for our analyses. Further, we separated the whole-brain WMH mask into the deep white matter hyperintensities (DWMH) and the periventricular white matter hyperintensities (PWMH). For segmentation, we used the widely accepted “continuity to ventricle” rule (Fazekas et al., 1987; Griffanti et al., 2018), which states that a WMH adjacent to the ventricle surface is PWMH, otherwise it is DWMH (Supplementary Figure 7).

### ROI selection

Although AD affects widespread areas, we selected specific deep gray matter (DGM) and cortical gray matter (CGM) regions (Supplementary Figure 1) based on strong evidence that they exhibit early and reproducible abnormalities in cerebral perfusion, oxygen utilization, and neurovascular function.

Within the DGM, the thalamus, putamen, and pallidum were chosen because these nuclei serve as major hubs within cortico-subcortical circuits and are consistently implicated in metabolic and neurovascular impairment across AD and related dementias (Tuokkola et al., 2019). The thalamus demonstrates reduced glucose metabolism and disrupted thalamo-cortical connectivity in both mild cognitive impairment and AD (Beason-Held et al., 2013; Jagirdar and Chin, 2019; Kumar et al., 2022; Schöll et al., 2011). Putamen and pallidum also show increased amyloid-beta plaque burden, along with perfusion abnormalities and neurovascular alterations (de Jong et al., 2008; Yang et al., 2023). Therefore, assessing OEF in these DGM regions provides biologically meaningful insight into subcortical contributions to impaired cerebral energetics in dementia.

Within the CGM, the rostral middle frontal gyrus (rostmidfront) and isthmus cingulate gyrus (isthcing), were selected because they represent cortical hubs where perfusion and metabolic disturbances are consistently demonstrated in AD. The rostmidfront, a key component of the executive-control network that supports working memory, attentional regulation, and higher-order cognitive control (Hertrich et al., 2021), has demonstrated hypoperfusion and reduced metabolic activity in MCI and AD (Sierra-Marcos, 2017). The isthmus cingulate gyrus forms part of the posterior default mode network (DMN)—one of the earliest and most robustly affected cortical systems in AD (Leech and Sharp, 2014). PET and fMRI studies have shown that the posterior cingulate and the adjoining cingulate isthmus display early hypometabolism, reduced cerebral blood flow and diminished connectivity with medial temporal structures in AD (Greicius et al., 2004; Minoshima et al., 1997). These characteristics make the rostmidfront and isthcing appropriate targets for assessing cortical oxygen-metabolism alterations within networks known to exhibit early vascular and metabolic changes in AD.

### Statistical analysis

**FIGURE 1:**
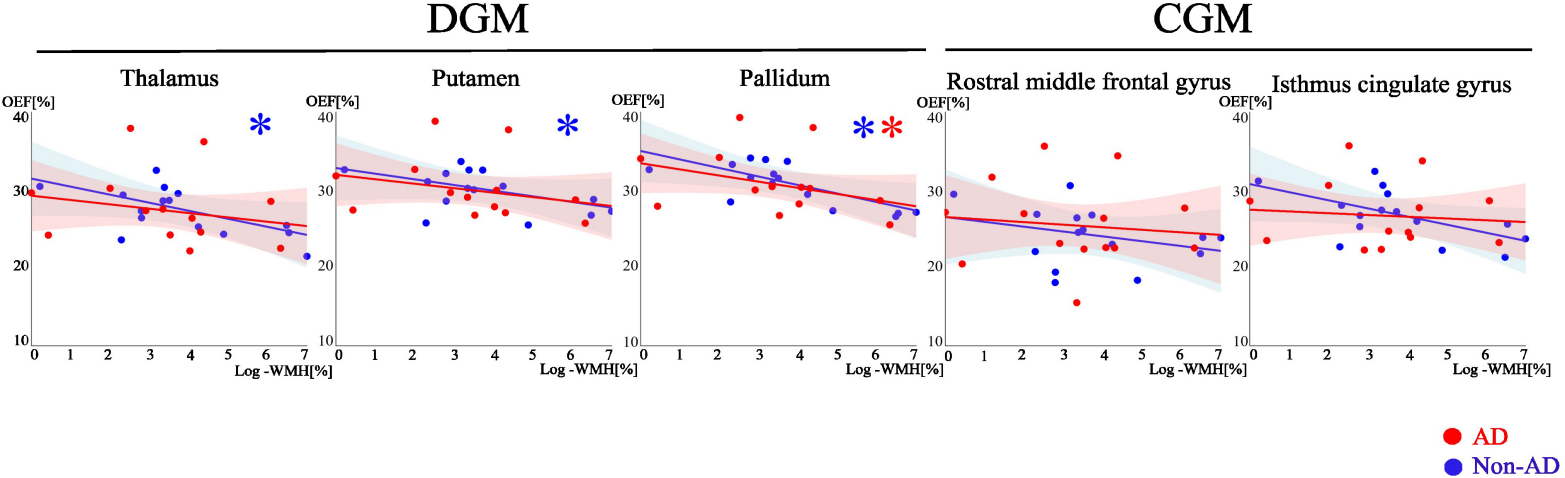
Scatter plots showing association between OEF and log-transformed WMH in DGM and CGM. Red and blue dots, line and shaded area represent data points, trend line and confidence intervals for AD and non-AD respectively. Multiple linear regression shows the significant (*p* < 0.05) negative trends in thalamus, putamen and pallidum, denoted by asterisk (*).

For the study cohort comparison between AD and non-AD (Table 1 and Supplementary Figure 2), Wilcoxon rank-sum test (for age, WMH and MoCA) and Fisher’s exact test (for binary variables, e.g., sex) were performed. To examine the associations between OEF with MoCA, we performed multiple linear regression analyses combining both groups, by taking OEF as the outcome variable, MoCA, group (AD = 1, non-AD = 0) and their interaction (e.g., MoCA*group) as independent variables, and age as a covariate. Because WMH exhibited a highly skewed distribution, WMH values were log-transformed prior to analysis. Associations between OEF and WMH were then examined using log(WMH), group, and their interaction term (log(WMH) × group) as independent variables, with age included as a covariate. To assess the regional specificity of these effects, the primary analyses were repeated using WMH subcomponents (PWMH and DWMH) as a sensitivity analysis. To assess the robustness of the regression results, influential and high-leverage data points were identified using (1) Cook’s distance with a threshold of 4/n, where n represents the number of observations, and (2) a leverage threshold of 2(p+1)/n, where p denotes the number of predictors in the model. Observed leverage values ranged from 0.161 to 0.663, and 5 observations exceeded the threshold. To further evaluate the impact of individual observations on specific regression coefficients, DFBETAs (standardized changes in regression coefficients are deletion of a single observation) were examined using a threshold of 2//√n (Supplementary Figure 3). Observations exceeding this threshold were flagged as potentially influential. Regression analyses were subsequently performed after excluding these influential data points (Figure 1). Residual diagnostics were also performed for OEF and MoCA (Supplementary Figure 4), which showed no systematic trends across ROI, indicating no violation of homoscedasticity assumptions. To account for multiple comparisons across multiple ROIs, the Benjamini-Hochberg correction was used to obtain adjusted (corrected) *p*-values (Benjamini and Hochberg, 1995). We reported the corrected *p*-values in this study, values less than 0.05 were considered statistically significant.

**TABLE 1:** Demographic and neuropsychological data of study subjects.

Variables	Alzheimer’s disease	Non-Alzheimer’s dementia	*P*-value
Number of patients	16	15	
Age (years)	69.18 (59–85) [7.87]	74.93 (57–91) [9.33]	0.07
Sex (Female: Male)	7 : 9	7 : 8	1
Whole brain OEF	24.84 (0.1072–0.3448) [0.0542]	24.87 (0.1973–0.303) [0.0309]	0.89
WMH	1.18 (1–2)	1.73 (1–3)	0.016 (*)
MoCA	18 (12–26)	20.45 (14–27)	0.045 (*)

Demographics and clinical characteristics of Alzheimer’s disease (AD) and Non-Alzheimer’s (non-AD) dementia. Data shown are means, (range), [standard deviations]. For WMH and MoCA, data shown as mean, (range). OEF is shown in percentage. *P*-value by Wilcoxon rank sum (for age, OEF, WMH and MoCA) and Fisher’s exact test (for sex). Asterisk (*) implies significant *p* values (< 0.05).

## Results

### OEF showed negative associations with WMH in the AD and non-AD group

**FIGURE 2:**
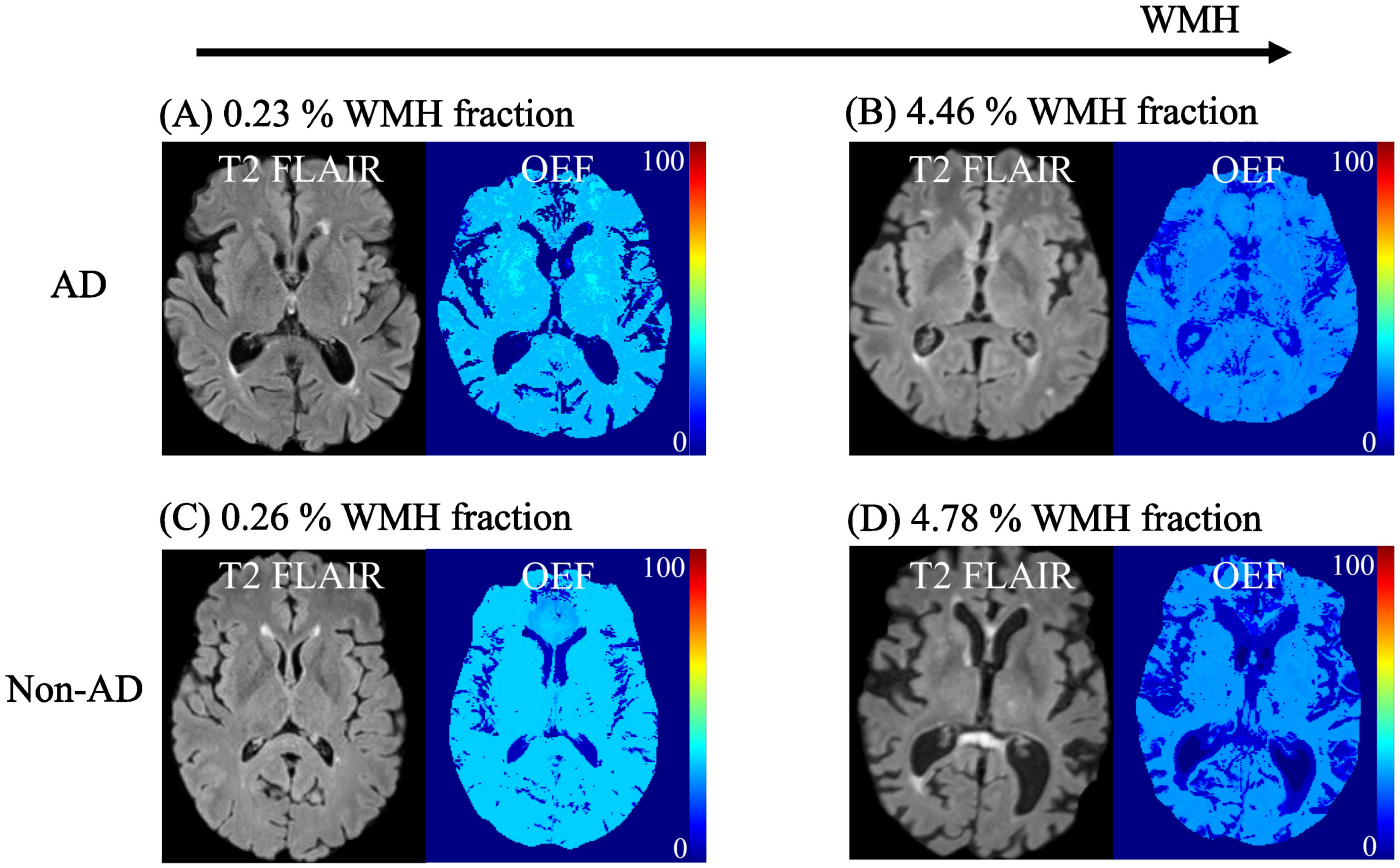
T2 FLAIR and OEF maps in Alzheimer’s disease (AD) and Non-Alzheimer’s (Non-AD) dementias. OEF decreases significantly with increasing WMH (log transformed) in pallidum in AD, from **(A)** to **(B)** and all of DGM in the non-AD group, from **(C)** to **(D)**.

We found negative associations between OEF and WMH in pallidum (p = 0.021) in the AD group and thalamus (*p* = 0.035), putamen (*p* = 0.03), and pallidum (*p* = 0.002) in the non-AD group (Figures 1, 2). No significant associations were observed in CGM.

### OEF showed positive associations with MoCA only in the AD group

**FIGURE 3:**
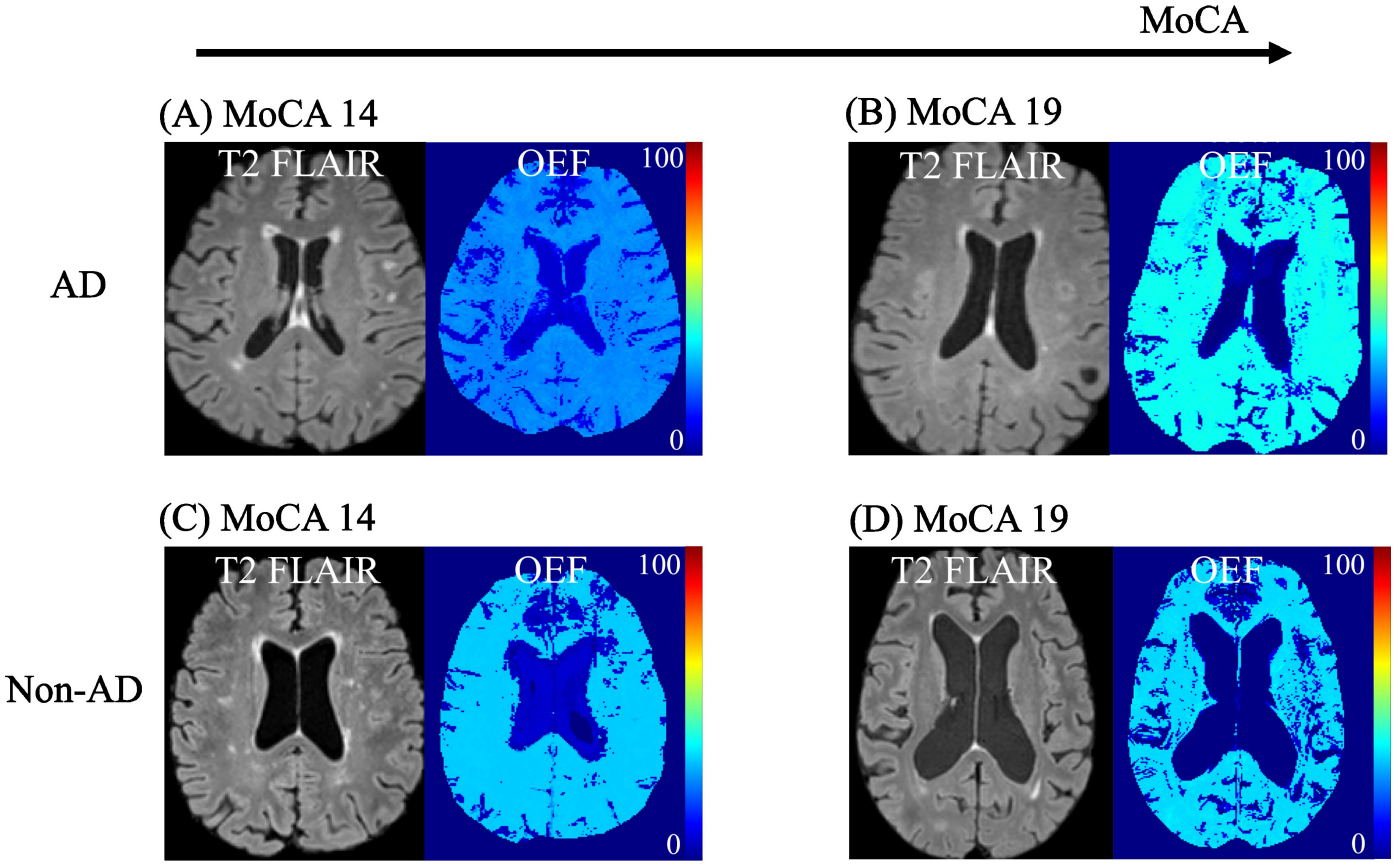
T2 FLAIR and OEF maps in AD and Non-AD dementias. OEF increases significantly with increasing MoCA in AD, from **(A)** to **(B)**. In the case of Non-AD, OEF does not significantly change as MoCA increases, from **(C)** to **(D)**.

**FIGURE 4:**
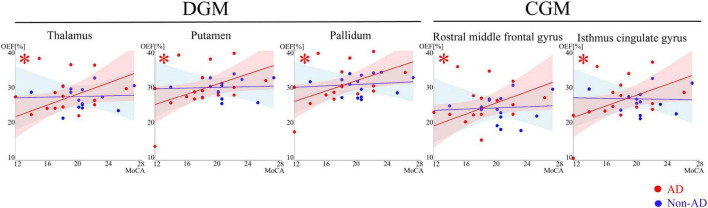
Scatter plots showing association between OEF and MoCA in DGM and CGM. Red and blue dots, line and shaded area represent data points, trend line and confidence intervals for AD and non-AD, respectively. Multiple linear regression shows the significant (*p* < 0.05) positive trends in thalamus, putamen and pallidum, rostral middle frontal gyrus and isthmus cingulate gyrus, denoted by asterisk (*).

OEF was positively associated with MoCA only in the AD group, both in DGM − thalamus (*p* = 0.049), putamen (*p* = 0.049), pallidum (*p* = 0.049) − and in CGM − isthcing (*p* = 0.049) and rostmidfront (*p* = 0.049) (Figures 3, 4 and Supplementary Figure 5).

## Discussion

This study demonstrated that QQ-based OEF mapping can identify oxygen metabolism-related pathophysiology in AD and non-AD dementia. We identified, for the first time, group specific associations between OEF with pathological biomarkers (e.g., white matter hyperintensity) and cognitive impairment measurement (MoCA). These findings suggest that QQ could provide new insights into the pathophysiology of AD and non-AD dementia.

First, we observed that lower OEF was associated with higher WMH only in DGM -pallidum in AD, and thalamus, putamen and pallidum in non-AD (Figure 1). These negative associations in AD patients align with prior studies (Arfanakis et al., 2020; Chiang et al., 2022; Yamaji et al., 1997), which show that WMH is linked to AD pathology in individuals with cognitive impairment. In AD, WMH is strongly associated with amyloid-beta deposition, tau-related neurodegeneration, microvascular damage, and axonal loss all of which can contribute to reduced OEF due to impaired neuronal and vascular coupling (Alban et al., 2023; Alber et al., 2019; Brickman and Rizvi, 2023; Garnier-Crussard et al., 2022; Laing et al., 2020; McAleese et al., 2021; Moscoso et al., 2020). The patterns observed in our non-AD group likely reflect cerebral small vessel disease (SVD). These diseases are thought to be the most frequent pathological processes and have a crucial role in non-AD dementia (Pantoni, 2010). WMH are a prominent feature of cerebral SVD (Gao et al., 2022; Wardlaw et al., 2017), reflecting chronic microvascular pathology which leads to cerebral hypoperfusion (Huang et al., 2025; Wolters et al., 2017). Chronic hypoperfusion associated with SVD limits oxygen delivery to brain tissue (Reiländer et al., 2023). Although compensatory mechanisms may initially attempt to maintain tissue oxygenation, progressive microvascular damage ultimately impairs the brain’s ability to extract and utilize oxygen efficiently. As a result, regional OEF decreases, reflecting a failure of oxygen delivery relative to metabolic demand. The negative associations between OEF and WMH observed in these regions in the non-AD group therefore support a predominantly vascular mechanism, in which WMH burden serves as a marker of underlying small vessel pathology and associated impairment of cerebral oxygen extraction. Although the patterns of association were observed separately in AD and non-AD groups, the interaction terms (e.g., MoCA × group) was not statistically significant, indicating no formal evidence of differential slopes between groups. Therefore, our findings should be interpreted as within group associations rather than definitive evidence of distinction between AD and non-AD.

The observation that this association is localized to DGM rather than CGM may be related to physiological differences between the two regions. Because DGM regions rely on deep perforating arteries with limited collateral blood flow, WMH-related microvascular damage can more directly and severely affects their oxygen extraction (Van Cauter et al., 2020; Wardlaw et al., 2015). Moreover, WMH primarily affects periventricular and deep white-matter tracts that directly project to or pass through DGM nuclei; hence, damage in these areas may directly influence DGM oxygen extraction (Lampe et al., 2019). In contrast, CGM is supported by a rich leptomeningeal collateral network that helps maintain blood flow even in the presence of microvascular injury, thereby reducing the impact of WMH on cortical oxygen delivery (Mangiardi et al., 2023). Additionally, in AD, cortical OEF may be affected by other major pathological processes−such as tau deposition, synaptic loss, and cortical thinning− that can overshadow any direct contribution of WMH to cortical oxygen-metabolism changes (Braak and Braak, 1991; Dickerson et al., 2009).

In our sensitivity analyses (Supplementary Figure 6), the negative association between OEF and WMH remained statistically significant in key DGM regions (except for the pallidum in the AD group), when comparing log-transformed WMH with untransformed WMH and when including or excluding influential observations. These analyses suggest that the observed association is robust and not driven by distributional skewness or a small number of high-influence data points. Furthermore, the inclusion of MCH as a covariate in our regression models did not alter the direction or statistical significance of these findings, suggesting that the observed associations are independent of microvascular burden.

When performing sensitivity analysis with regional WMH (Supplementary Figure 8), the results remained similar between OEF and log-transformed PWMH. No associations were observed with DWMH. These findings suggest that the observed associations are mainly driven by PWMH rather than DWMH.

Second, in AD, as the MoCA score increases, the regional OEF increases in DGM (e.g., thalamus, putamen, and pallidum) and CGM (isthcing and rostmidfront) (Figure 4). Since higher MoCA scores indicate better cognitive performance (Carson et al., 2018), this positive correlation between MoCA and OEF suggests that better cognitive function is associated with more efficient oxygen utilization in these regions. This finding aligns with previous studies showing a positive relationship between OEF and information processing speed in patients with neurologic disorders (Eizaguirre et al., 2018; Li et al., 2023), particularly with one study reporting that elevated OEF was positively associated with composite cognitive score in patients with mild cognitive impairment (Jiang et al., 2020). Together, these results and the positive association between MoCA and OEF found in our study suggest that maintaining efficient oxygen metabolism may be vital for preserving cognitive function in AD patients. Although the interaction term did not reach statistical significance, indicating that we cannot confirm a statistically divergent slope between groups, we observed robust within-group associations uniquely within the AD cohort.

Conversely, the absence of such relationships in non-AD dementias suggests that cognitive impairment in these cases may be driven by other mechanisms, such as vascular insufficiency, diffuse white matter disconnection, or widespread brain network dysfunction, which may not depend on efficient oxygen extraction to the same extent. Unlike AD—where metabolic compromise is closely tied to cognitive decline—non-AD dementias may involve pathophysiological pathways that are less reliant on oxygen extraction capacity.

## Limitation

There are several limitations to the present study. First, the small sample size restricts the scope of our analyses, preventing the exploration of dementia subtypes and treatment effects. It also limits the number of regions that could be included in our analysis due to reduced statistical power. Consequently, other brain regions relevant to AD pathology, such as hippocampus and medial temporal regions, were not included among the selected ROIs in this study. Their metabolic alterations warrant further investigation in larger cohorts. Additionally, the absence of a healthy control group limits our ability to determine whether the observed associations are specific to dementia or reflect general neurodegeneration processes.

Second, the assumptions underlying QQ modeling may not fully apply to AD pathology, potentially introducing biases in OEF estimation. For instance, QQ modeling assumes a randomly oriented distribution of numerous blood vessels (Cho et al., 2018). Future research is necessary to validate this assumption in AD, particularly if significant microvascular alterations are present, as these would need to be incorporated into QQ modeling. Additionally, QQ modeling does not account for the detailed microstructure of neural tissue, such as myelin, which could influence OEF estimation. Given the demyelination and neuronal loss commonly observed in AD, these factors may hinder accurate comparisons of OEF. QQ modeling assumes that deoxyhemoglobin in venous structures and diffusely distributed non-blood tissue components (e.g., ferritin or proteins) are the two primary compartments within a voxel. However, if other structured sources, such as myelin, contribute to intravoxel field variations, inaccuracies in QQ-based OEF estimation could arise. Despite this, our previous QQ validation study showed no significant differences in OEF between QQ and ^15^O-PET (the reference standard) in white matter (Cho et al., 2021a), suggesting that the impact of myelin on QQ-based OEF estimation is likely insignificant. Furthermore, OEF estimation in this study assumed a fixed hematocrit (Hct) value of 0.3567 based on established literature (Zhang J. et al., 2018). However, Hct may vary in AD and dementia due to aging, anemia, and other vascular comorbidities (Hong et al., 2013; Wolters et al., 2019). Because susceptibility-based OEF estimation depends on hemoglobin concentration, variability in Hct may introduce bias when a fixed Hct value is assumed (e.g., if true Hct is higher than the assumed Hct, the QQ model may overestimate OEF). Additionally, iron deposition could be a potential confound in OEF estimation. However, in principle, the QQ model separates the OEF effect from neural tissue susceptibility (χ_n_, which is mainly derived from diffusive iron in tissue. Consequently, pathological iron deposition is reflected in χ_n_ estimation, rather than OEF. Our previous study (Chiang et al., 2022) showed significantly higher χ_n_ in AD patients compared to healthy controls, suggesting that pathological iron deposition in AD is adequately accounted for in the QQ model through χ_n_ estimation and is therefore unlikely to introduce a significant bias in OEF estimation.

Third, our study cohort lacked extensive neurophysiological testing to assess cognitive function. Moreover, it was unclear where our non-AD patients have underlying vascular disease/risk factor or not, which limited our ability to conduct a detailed comprehensive analysis of dementia subtypes.

Fourth, the cross-sectional design of this study restricted our capacity to assess differences in disease progression between the AD and non-AD cases over time. Future longitudinal studies are warranted to elucidate temporal changes in OEF and their relationship with cognitive decline. Moreover, the use of region specific WMH might be a scope for further studies.

Fifth, macroscopic field correction may not be fully corrected by the VSF method used in this study, which could introduce bias in OEF estimation. To quantify residual field effects, we compared OEF estimates under three preprocessing conditions: (i) default VSF correction using nearest neighboring voxels (as used in this study), (ii) higher-order VSF (using a 3 × 3 × 3 neighborhood), and (iii) no VSF correction. Each variation was applied independently to each subject, and whole-brain OEF was computed for each subject prior to group-level statistical analysis.

The resulting whole-brain average OEF values were 25.7% ± 10.2 with default VSF, 26.9% ± 8.9 without VSF, and 25.6% ± 10.5 with higher-order VSF. The 1.2% decrease in OEF with default VSF suggests that VSF effectively removes signal decay caused by macroscopic field inhomogeneities and reduces OEF overestimation. Although applying a higher-order VSF provided an additional 0.1% reduction in OEF, the differences were not statistically significant (*p* = 0.57, paired *t*-test), suggesting that the default VSF correction effectively reduces macroscopic field effects. In addition, the clustering and total variation regularization in the CCTV algorithm used in this study are expected to further mitigate the influence of macroscopic fields on OEF estimation. However, in regions with substantial susceptibility gradients (e.g., voxels adjacent to the nasal cavities), residual macroscopic fields may persist and affect OEF estimation. Further studies are warranted to rigorously investigate the impact of strong regional macroscopic field effects. In addition, although OEF estimation was not highly sensitive to the choice of different background field removal algorithm (e.g., OEF was 25.70 ± 10.20% for PDF and 25.72 ± 8.30% *p* = 0.22, Wilcoxon rank-sum test), a rigorous and systematic simulation-based assessment of the impact of different background field removal and macroscopic field correction strategies on OEF estimation is warranted.

Sixth, macroscopic field inhomogeneities were compensated using signal-specific models (e.g., PDF for phase signals and VSF for magnitude signals) rather than a unified framework. Although both PDF and VSF assumes smoothly varying background fields that are distinct from local field and are therefore expected to provide similar macroscopic field compensation for magnitude and phase signals, residual macroscopic fields may differentially bias QSM- and qBOLD-related components in the combined QQ modeling, which could affect OEF estimates. Future work is warranted to investigate the impact of distinct macroscopic field compensation strategies on OEF estimation.

Seventh, to evaluate the inter-rater reliability on WMH masking, we performed independent manual WMH segmentation on a random subset of five cases with relatively high WMH by an experienced neuroradiologist (Dr. M. Reza Taheri) and calculated the DICE Similarity Coefficient (DSC) (Zou et al., 2004). The analysis yielded a mean DSC of 0.58 ± 0.11 (range: 0.42 – 0.75), which falls within the previously reported range of 0.55-0.75 in aging and neurodegenerative cohorts (Kuijf et al., 2019), where WMHs are often small or sparse. However, this reliability analysis was performed on a limited subset of cases, and more rigorous and comprehensive inter-rater WMH segmentation assessments are warranted in future studies.

Lastly, although within group associations were observed, the interaction terms were not statistically significant. Larger cohorts may be required to determine whether these trends represent true between-group differences or if our study was underpowered to detect subtle interaction effects.

In conclusion, our study demonstrated the feasibility of QQ for understanding oxygen-related pathophysiology in AD and non-AD dementia, potentially leading to improved treatment strategies for both.

## Data Availability

The raw data supporting the conclusions of this article will be made available by the authors, without undue reservation.
