# Supplementary material for: Oxygen extraction fraction is differentially associated with pathological biomarkers in Alzheimer’s disease and non-Alzheimer’s dementias
Source: Front Neurosci. 2026 Apr 28;20:1754415. doi: 10.3389/fnins.2026.1754415 (PMC13161180; doi:10.3389/fnins.2026.1754415)

**
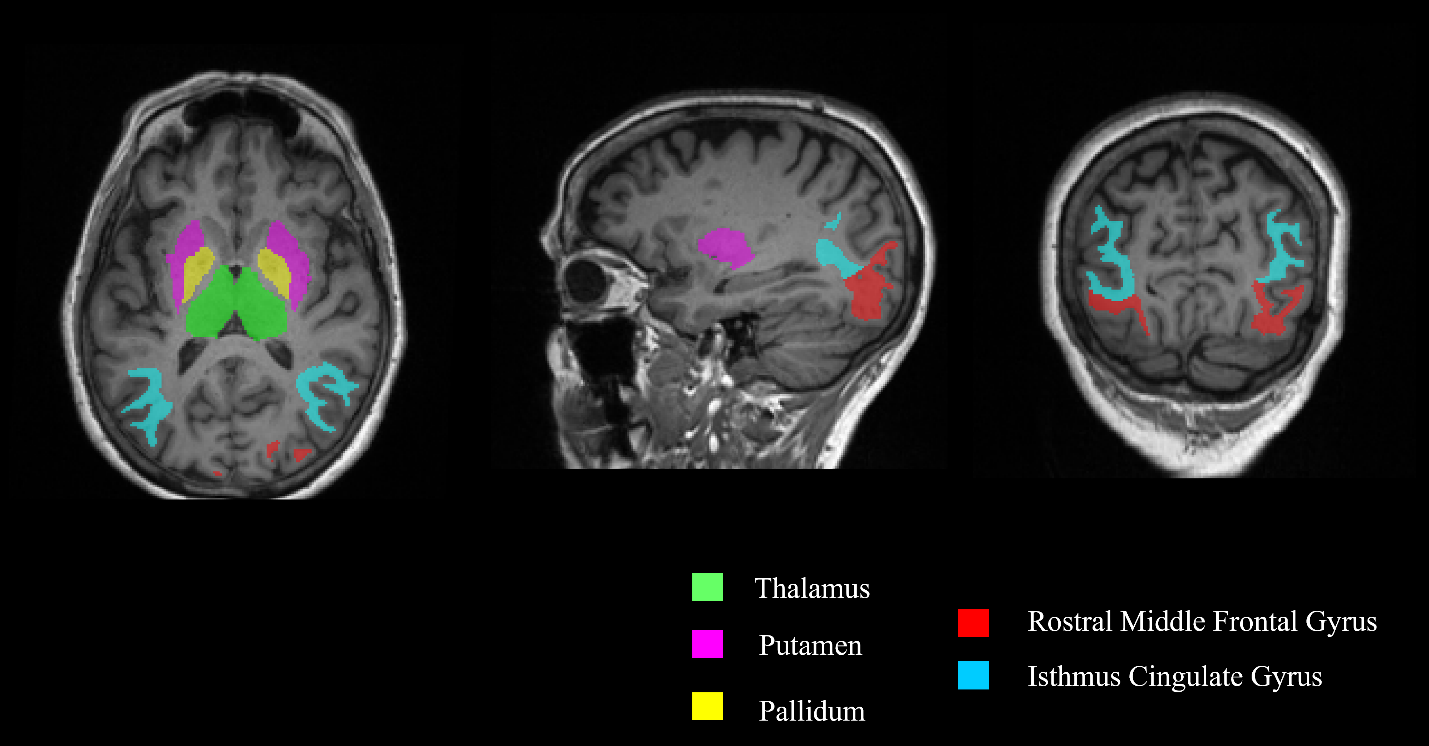
Supplemenary Figure 1:** Axial, sagittal and coronal (from left to right) representation of an AD patient. Regions are noted in the color-coded key.


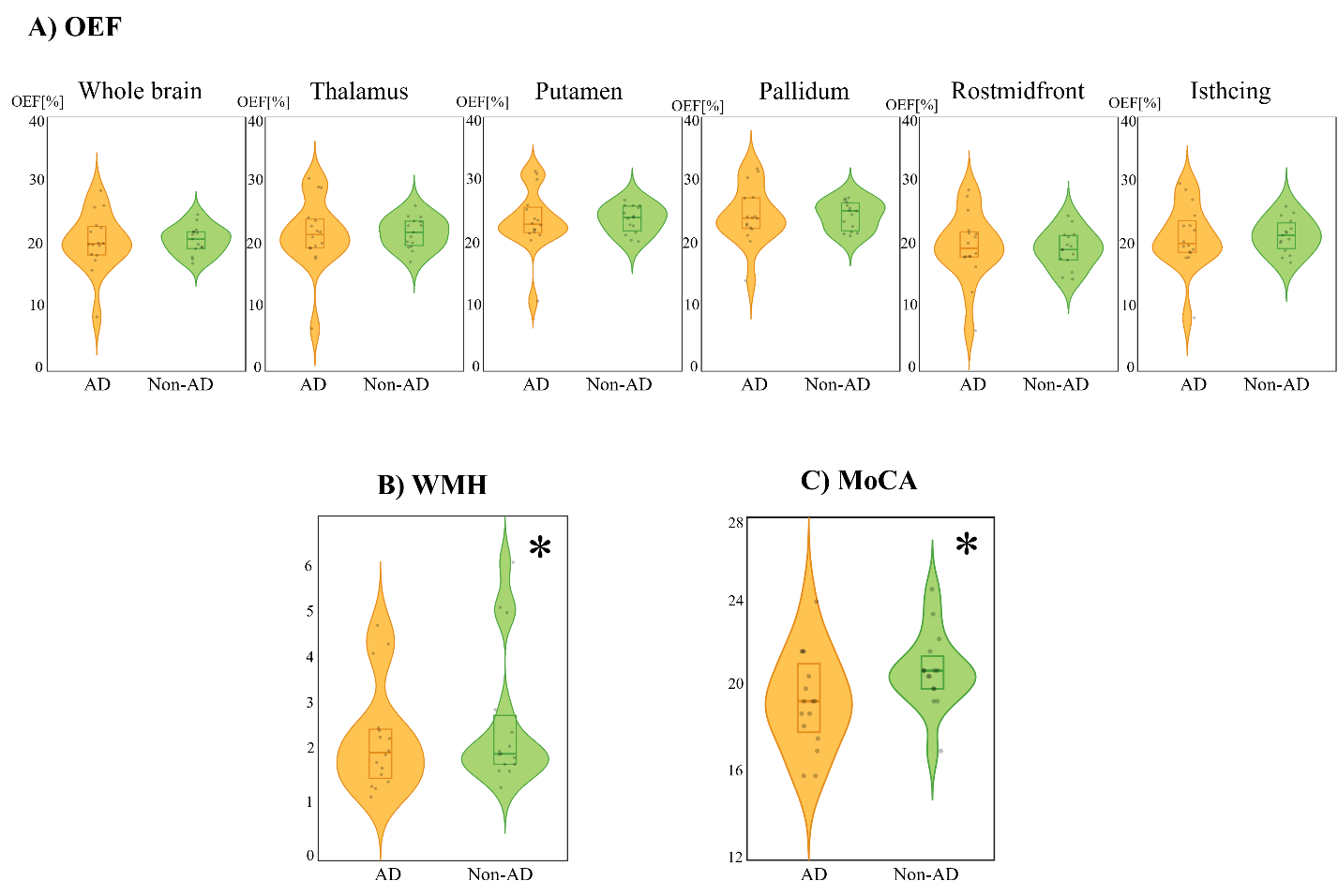
**Supplemenary Figure 2:** Comparison betweem AD and non-AD groups: (A) OEF, (B) WMH, and (C) MoCA. Asterisks (*) denote statistically significant differences based on the Wilcoxon rank-sum test (p<0.05).

**Supplementary Figure 3:** Residual diagnostics from OEF vs log (WMH). (A) residual vs. fitted plots, (B) Cook’s distance D and DFBETAs (standardized changes in regression coefficients after the deletion of a signal observation).


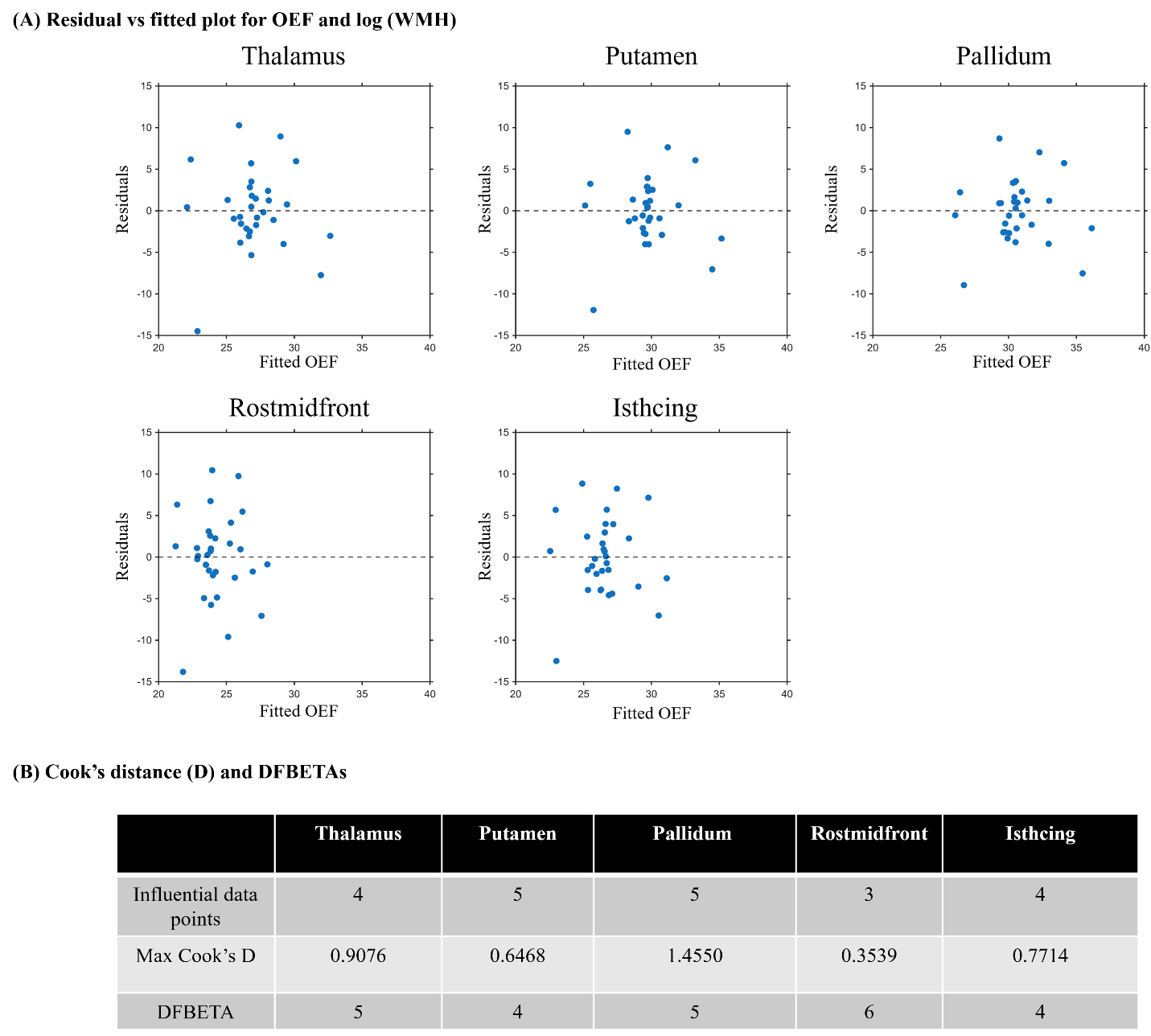


**Supplementary Figure S4:** Residual vs. fitted plots from OEF vs. MoCA.


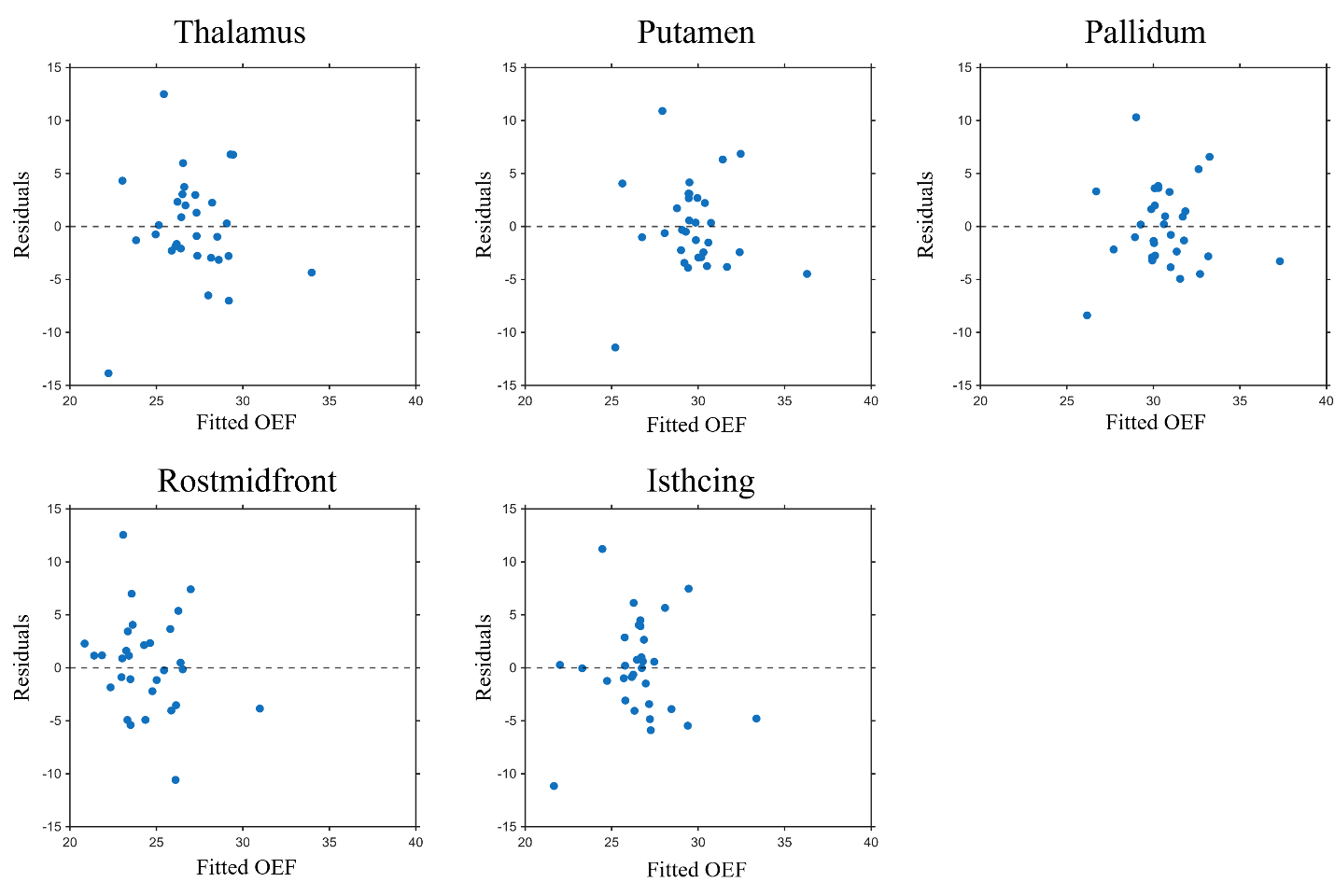


**Supplementary Figure 5:** Linear regression results examining the association between OEF and MoCA, adjusted for age, in the selected regions of interest (ROIs) in (A) non-AD and (B) AD groups. Slopes (β), standard errors (SE), t-statistics (t), uncorrected p-values, Benjamini–Hochberg false discovery rate–adjusted p-values (BH-q), and 95% confidence intervals (CI) are reported separately (taking OEF as percentage).

*
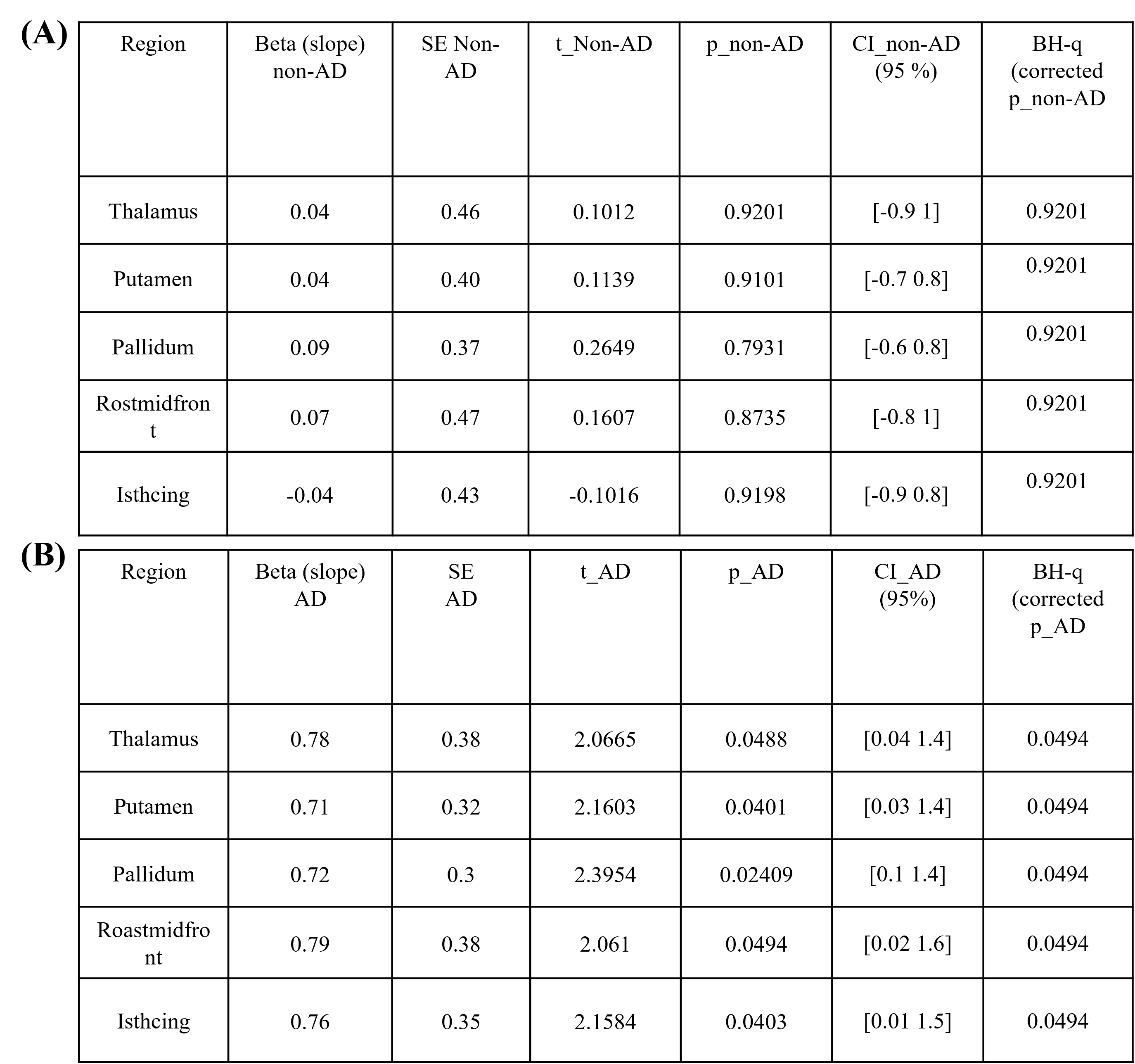
*

**
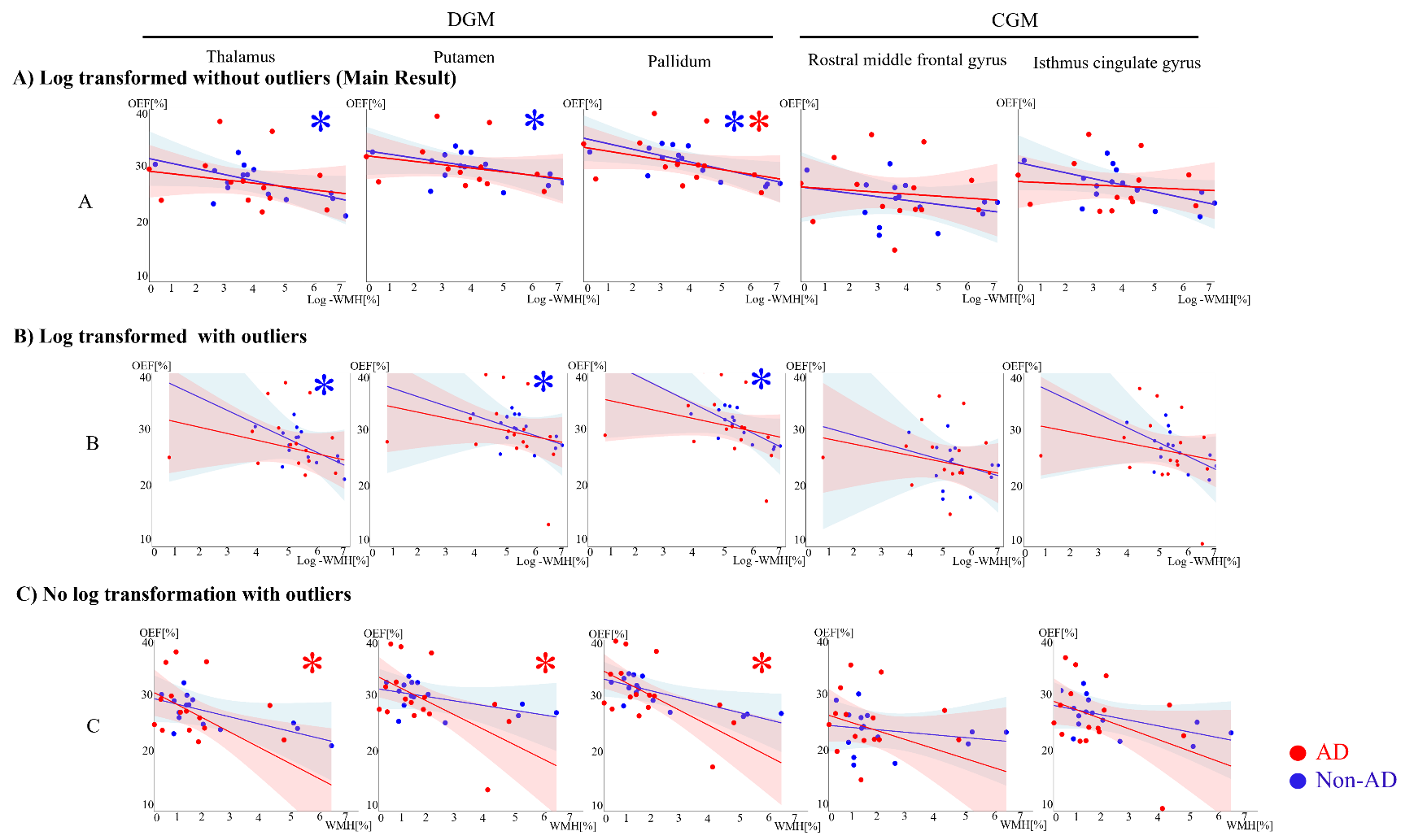
Supplementary Figure 6:** Scatter plots showing associations between OEF and WMH in DGM and CGM. A and B represent association with log transformed WMH – without outliers (A; main result) and with outliers (B). C represents association without log transformed WMH and OEF. Red and blue dots, line and shaded area represent data points, trend line and confidence intervals for AD and non-AD respectively. Asterisks (*) denote statistically significant associations using multiple linear regression (p<0.05).

**Supplementary Figure 7:** Example of WMH sub classification obtained from T2 FLAIR images. PWMH – periventricular WMH and DWMH – deep WMH.


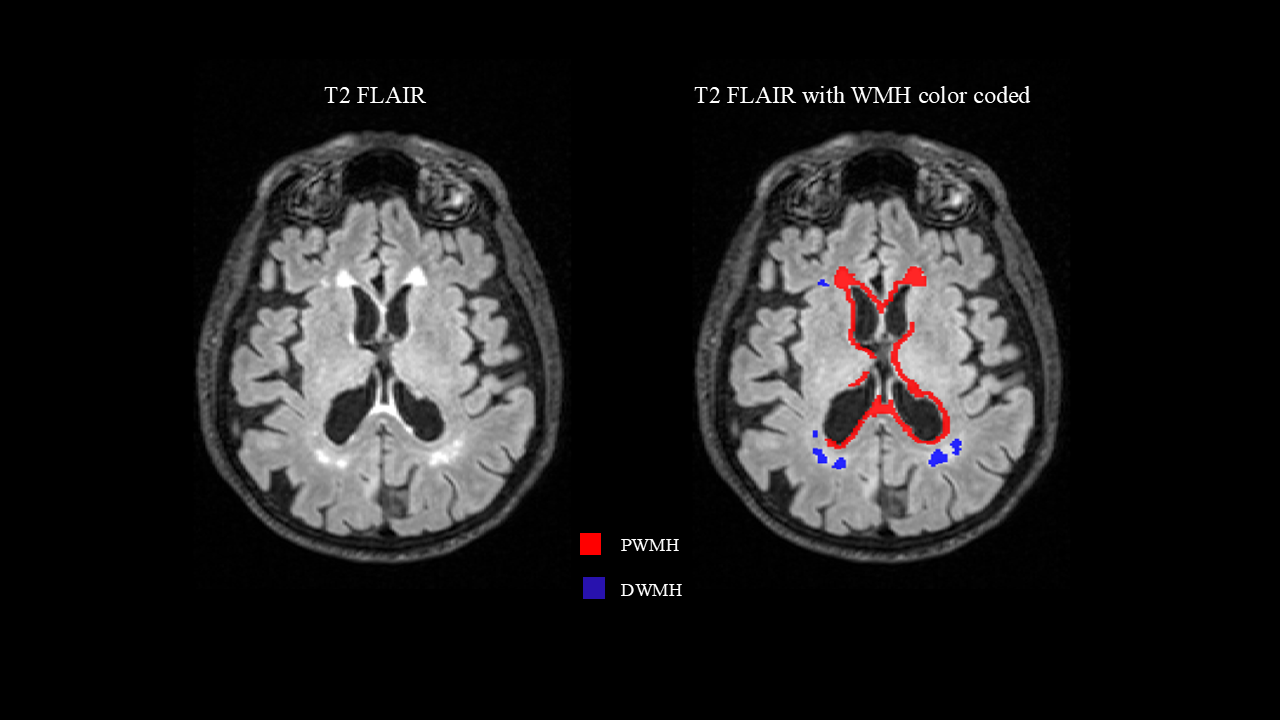


**Supplementary figure 8:** (A) Boxplots for comparison of total WMH, periventricular WMH (PWMH), and deep WMH (DWMH). P value by Wilcoxon rank-sum test. (B) Raincloud plot illustrating the regional burden of total WMH, PWMH, and DWMH across the study cohort. Each panel includes a kernel density estimate ("cloud") showing the probability distribution, a horizontal boxplot representing the median and interquartile range, and individual jittered data points ("rain") representing WMH percentage compared to the whole brain.


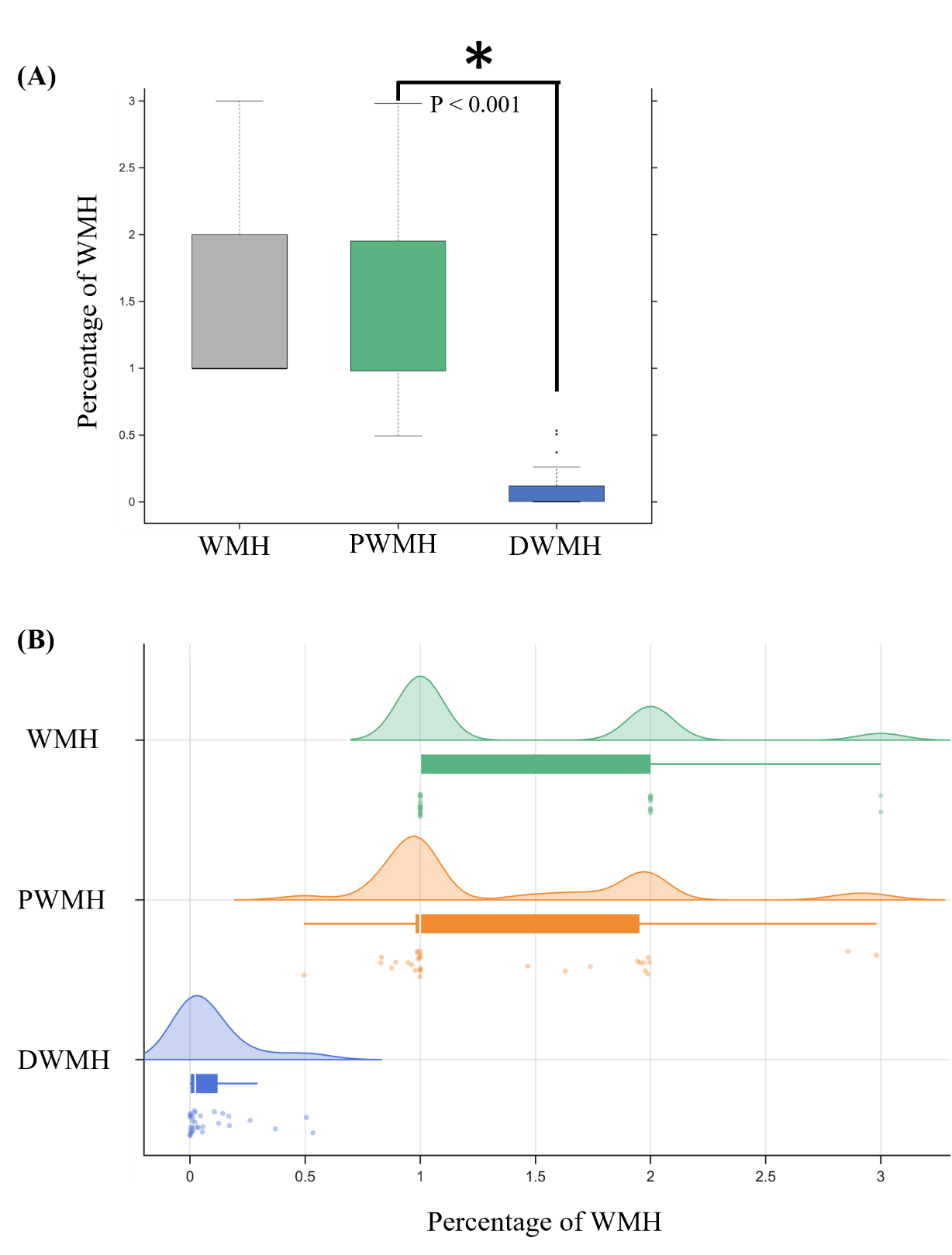

Supplement: Supplementary file 1 [file Data_Sheet_1.docx]
